# Latent Fairness in Adults’ Relationship-Based Moral Judgments

**DOI:** 10.3389/fpsyg.2015.01871

**Published:** 2015-12-10

**Authors:** Jian Hao, Yanchun Liu, Jiafeng Li

**Affiliations:** ^1^Beijing Key Laboratory of Learning and Cognition, Department of Psychology, College of Education, Capital Normal UniversityBeijing, China; ^2^Youth Work Department, China Youth University of Political StudiesBeijing, China; ^3^Psychological Education and Counseling Center, Office of Student Affairs, Yunnan University of Finance and EconomicsKunming, China

**Keywords:** moral judgments, relationships, fairness, familiarity, affective relatedness

## Abstract

Can adults make fair moral judgments when individuals with whom they have different relationships are involved? The present study explored the fairness of adults’ relationship-based moral judgments in two respects by performing three experiments involving 999 participants. In Experiment 1, 65 adults were asked to decide whether to harm a specific person to save five strangers in the footbridge and trolley dilemmas in a within-subject design. The lone potential victim was a relative, a best friend, a person they disliked, a criminal or a stranger. Adults’ genetic relatedness to, familiarity with and affective relatedness to the lone potential victims varied. The results indicated that adults made different moral judgments involving the lone potential victims with whom they had different relationships. In Experiment 2, 306 adults responded to the footbridge and trolley dilemmas involving five types of lone potential victims in a within-subject design, and the extent to which they were familiar with and affectively related to the lone potential victim was measured. The results generally replicated those of Experiment 1. In addition, for close individuals, adults’ moral judgments were less deontological relative to their familiarity with or positive affect toward these individuals. For individuals they were not close to, adults made deontological choices to a larger extent relative to their unfamiliarity with or negative affect toward these individuals. Moreover, for familiar individuals, the extent to which adults made deontological moral judgments more closely approximated the extent to which they were familiar with the individual. The adults’ deontological moral judgments involving unfamiliar individuals more closely approximated their affective relatedness to the individuals. In Experiment 3, 628 adults were asked to make moral judgments with the type of lone potential victim as the between-subject variable. The results generally replicated those of the previous two experiments. Therefore, the present study shows that, in addition to apparent unfairness, latent fairness exists in adults’ relationship-based moral judgments. Moral judgments involving individuals with whom adults have different relationships have different cognitive and affective bases.

## Introduction

Society born and society bred, all individuals are inevitably confronted with various moral problems. How these problems are solved crucially depends on one’s moral judgment. Moral judgments refer to evaluations of behavior in terms of right and wrong (Haidt, 2001). It has been demonstrated that adults’ moral judgments play an important role in their morals-related behavior, such as prosocial, delinquency, and cheating behavior (Raaijmakers et al., 2005; Carlo et al., 2012; Xu and Ma, 2015).

Moral judgments are typically reflected in one’s responses to moral dilemmas. In the classic trolley dilemma (Thomson, 1985), a runaway trolley is about to hit five people standing on its track. The only way to save the people is to switch the trolley onto another track. However, the intervention will cause the death of one person standing on the other track. Should the one person be harmed to save the five people? Different principles support different decisions. The utilitarian principle (Mill, 1998) emphasizes that it is important to maximize the good of the majority of people. Therefore, switching the trolley to save the five people is the right choice. By contrast, according to the deontological principle (Kant, 1959), behavior must be in accordance with moral rules regardless of the consequences. From this perspective, redirecting the trolley onto another track is unacceptable because it is immoral to harm an innocent person. Previous studies show that most adults choose to save the five people by harming the lone person, making utilitarian moral judgments (Hauser et al., 2007; Waldmann and Dieterich, 2007; Pellizzoni et al., 2010; Navarrete et al., 2012).

However, adults seem to abide by the utilitarian principle only in the case of the trolley dilemma. In a variant of the dilemma, the footbridge dilemma (Thomson, 1986), a large person on a footbridge can be pushed down to prevent a trolley from hitting five people. At the same time, the intervention will undoubtedly cause the death of the large person. When physical contact is involved, adults less often choose to save more people at the cost of one person’s life and judge that this type of harm is worse than harm involving no physical contact (Cushman et al., 2006; Hauser et al., 2007; Pellizzoni et al., 2010; Powell et al., 2012).

The less utilitarian choice in the footbridge dilemma implies that adults do not consider the good of five people to always be more important than that of one person. Specifically, in the footbridge dilemma, adults are rather close to the lone potential victim. The close spatial distance may cause adults to pay more attention to the welfare of the person. Is it possible that mental distance, i.e., relationships, between adults and the lone potential victim also affects their moral judgments? Previous studies provide some indirect or incomplete evidence. For example, Ma (1992) found that adolescents and adults showed a greater altruistic orientation toward others with whom they had closer relationships. Similarly, Bleske-Rechek et al. (2010) discovered that the more adolescents and adults were genetically related to the lone potential victim, the less they were willing to sacrifice the person’s life in the trolley dilemma. A recent study (Tassy et al., 2013) examined students’ moral judgments involving relatives, close friends and strangers and found that their moral judgments depended on their affective proximity to these people.

Although previous studies have examined the effect of relationships on adults’ moral judgments to some extent, they have mainly focused on positive relationships. According to the hierarchy of human relationships (Ma, 1985, 1992), people can be categorized into different groups including relatives, best friends, special strangers, common strangers and someone you dislike or enemies in order. An actor has the strongest altruistic orientation toward the group ranking first and the weakest altruistic orientation toward the group ranking last because the actor has different relationships with these groups of people. The relationships vary in genetic relatedness, familiarity, and affective relatedness. As a result, it is necessary to include lone potential victims who have different relationships with adults in terms of these three dimensions.

More importantly, it remains unclear whether adults’ relationship-based moral judgments reflect the unfairness of their moral judgments. Although previous studies have shown that unfairness exists in adults’ moral judgments, the studies appear inconsistent with some important theoretical perspectives. In cognitive-developmental theory, moral judgments eventually reach the post-conventional level (Colby and Kohlberg, 1987). At this level universal principles of justice are important in one’s moral judgments. One cares about the equality of all people’s rights. According to moral foundations theory (Haidt and Joseph, 2004; Haidt and Graham, 2007), some psychological systems evolve and lead to certain intuitive foundations in the moral domain across cultures. One of the foundations is that of fairness/reciprocity. Fairness develops because specific emotions such as guilt evolve and are experienced in reciprocal interactions. Therefore, fairness may exist in adults’ moral judgments according to these theoretical perspectives. Taken together, empirical studies and classic theories suggest that there may be different aspects of fairness in adults’ moral judgments. The fairness of their moral judgments may be manifested in one aspect but not others. In fact, there may be at least two aspects of fairness. One emphasizes whether different individuals are treated fairly. This aspect involves cross-sectional comparisons of adults’ moral judgments toward different individuals. It appears that adults treat different types of lone potential victims differently and thus apparent unfairness exists in their moral judgments. The other emphasizes whether an individual is treated fairly relative to his/her relationships with decision-makers. This aspect involves longitudinal comparisons of adults’ moral judgments toward the individual and their relationships with the individual. If adults’ moral judgments involving specific individuals correspond to their relationships with the individuals, latent unfairness exists in their moral judgments. For example, some adults believe that they very much should not protect the lone potential victim when they are very unfamiliar or very much dislike the person. However, consider the following example. Although adults are not familiar with or do not like a potential victim, they are still willing to protect the person to some extent, which indicates that the extent to which adults protect the person does not match the extent to which they are familiar with or like the person. Adults make deontological moral judgments involving the person to a greater extent relative to their relationships with the person. Therefore, there is latent fairness in adults’ moral judgments.

Furthermore, the importance of cognition and affect in adults’ relationship-based moral judgments remains unclear. According to the perspectives of Greene et al. (2001, 2004), moral judgments are driven by either cognitive or emotional processes. The researchers also maintain that the two processes are sometimes competitive. Because the extent to which adults are familiar with or like specific individuals varies, it is possible that cognitive processes play a crucial role in adults’ moral judgments involving certain types of individuals, whereas affective processes are important for their moral judgments involving other types of individuals. Tassy et al. (2013) indicated that students’ moral judgments were influenced by their affective proximity to the potential victim, but how they were affectively related to different potential victims was not investigated. Therefore, it is necessary to measure the extent to which adults are familiar with and like the lone potential victim. If adults’ moral judgments approximate their familiarity with the lone potential victim, the cognitive effect is important. If their moral judgments approximate their affective relatedness to the lone potential victim, the role of affect is distinct.

In sum, the present study aims to explore the fairness of adults’ relationship-based moral judgments and its cognitive and affective bases. In Experiment 1, adults were asked to decide whether to harm a specific individual to save five strangers in simplified footbridge and trolley dilemmas. The potential victims differed in whether they were genetically related to, familiar to and liked by the adults. In Experiment 2, the adults’ moral judgment pattern found in Experiment 1 was tested in the classic footbridge and trolley dilemmas with a large sample. The extent to which adults were familiar with and liked the potential victim was measured, and its correspondence to their moral judgments was examined. Experiment 3 further tested previous results with the type of lone potential victims as the between-subject variable. Because the principle of fairness develops in adulthood, it is hypothesized that there may be latent fairness in adults’ relationship-based moral judgments. Adults’ moral judgments involving close individuals may be less deontological relative to their positive relationships with these individuals. Adults’ moral judgments involving individuals they are not close to may be more deontological relative to their negative relationships with these individuals. Furthermore, the latent fairness may also make cognition and affect differ in their importance for relationship-based moral judgments. Adults’ deontological moral judgments involving familiar individuals may more closely approximate their familiarity with these individuals because familiarity is a more objective basis. For individuals with whom adults are not familiar, affect may be the only available basis for their moral judgments. Thus, adults’ deontological moral judgments may more closely approximate their affective relatedness to these individuals.

## Experiment 1

The aim of Experiment 1 was to reveal adults’ moral judgment pattern involving individuals with whom they had various relationships. Adults were required to decide whether to harm a specific individual to save five strangers in simplified footbridge and trolley dilemmas. The lone potential victim was a relative, a best friend, a person whom the adult disliked or a criminal, with a stranger as the control condition.

### Participants

Sixty-five college students participated in the experiment: 31 males and 34 females. Their ages ranged from 18.92 to 27.67 years (*M* = 20.71, *SD* = 1.58). In a within-subject design, a footbridge and a trolley dilemma were presented to each participant. Participants were asked to answer five test questions in each dilemma. A within-subject design was used to indicate whether one participant made fair judgments when different lone potential victims were involved. A stranger-conditioned test question was asked first because participants’ answer to it served as their baseline performance. The other test questions were presented in a fixed order because our pilot experiment found that participants gave consistent answers regardless of the question order. The experiment was approved by the Research Ethics Board of Department of Psychology of Capital Normal University. Informed written consent was obtained from all of the participants.

### Materials and Procedure

#### The Lone Potential Victim

Based on the hierarchy of human relationships (Ma, 1985, 1992), the lone potential victim in the experiment was a relative, a best friend, a person whom the participant disliked, or a criminal. Participants were closest to their relatives and most distant from criminals in terms of relationships. Because a common stranger had no specific relationship with participants, the stranger was regarded as the control condition. The relationships between participants and each lone potential victim are presented in **Table 1**.

**Table 1:** The relationships between participants and each lone potential victim.

The potential victim	Genetic relatedness	Familiarity	Affective relatedness
Stranger	Genetic unrelated	Unfamiliar	Neutral
Relative	Genetic related	Familiar	Positive
Best friend	Genetic unrelated	Familiar	Positive
Disliked person	Genetic unrelated	Familiar	Negative
Criminal	Genetic unrelated	Unfamiliar	Negative

#### Moral Judgments

The footbridge and trolley dilemmas of Pellizzoni et al.’s (2010) were used to measure participants’ moral judgments. The dilemmas were tested in samples of children and adults. The scenarios were relatively simple, and control questions guaranteed participants’ correct understanding of the scenario in each dilemma; thus, participants could understand the dilemmas without the interference of many details. A minor revision that was made in the present experiment was that participants served as the intervention actors in the two dilemmas. For example, in the original footbridge dilemma, the intervention actor was a protagonist John. In the present experiment, the intervention actors were the participants. Therefore, “John” was replaced by “you” in the moral dilemma below.

Participants were tested individually in a quiet room. The experimenter described the moral dilemmas to the participants. At the same time, the participants were presented with a schematic illustration of each moral dilemma. Thus, participants could directly witness the moral situations that were described and understand them well. They then answered the control questions and test questions involving lone potential victims with whom they had different relationships.

The footbridge dilemma (Pellizzoni et al., 2010, p. 266)

A big ball is running quickly along this track. On the track, there are five people who do not see the ball rolling down because they are looking in front of them. (Control question 1: Do these people see the ball rolling down the street?) The ball is rolling down this street and it will soon badly hurt five people. You are on a footbridge between the big ball and the five people. On the footbridge, close to you there is a big person. The person does not see the ball rolling down because he is looking in front of him. (Control question 2: Does this person see the ball rolling down?) You know that the only way to stop the big ball is to drop a very heavy weight into the street. You know that if you push down the big person close to you, the big person will be badly hurt but the other five people will be safe. Test question 1: What should you do? Push the person or not push the person?

Test questions 2–5 were the same as question 1 except that the lone potential victim was a relative, a best friend, a person whom the participant disliked and a criminal, respectively. For example, test question 2 was “If the person is your relative, push the person or not push the person?” Therefore, in the footbridge dilemma, the lone potential victim was the one to push in the footbridge.

The trolley dilemma (Pellizzoni et al., 2010)

The trolley dilemma resembled the footbridge dilemma except that the participants could pull a string and make the ball go onto another track where one person stood. Accordingly, the intervention would badly hurt the person. Participants were asked similar control and test questions. Therefore, in the trolley dilemma, the lone potential victim was the one who stood alone on the alternative track.

### Results and Discussion

**FIGURE 1:**
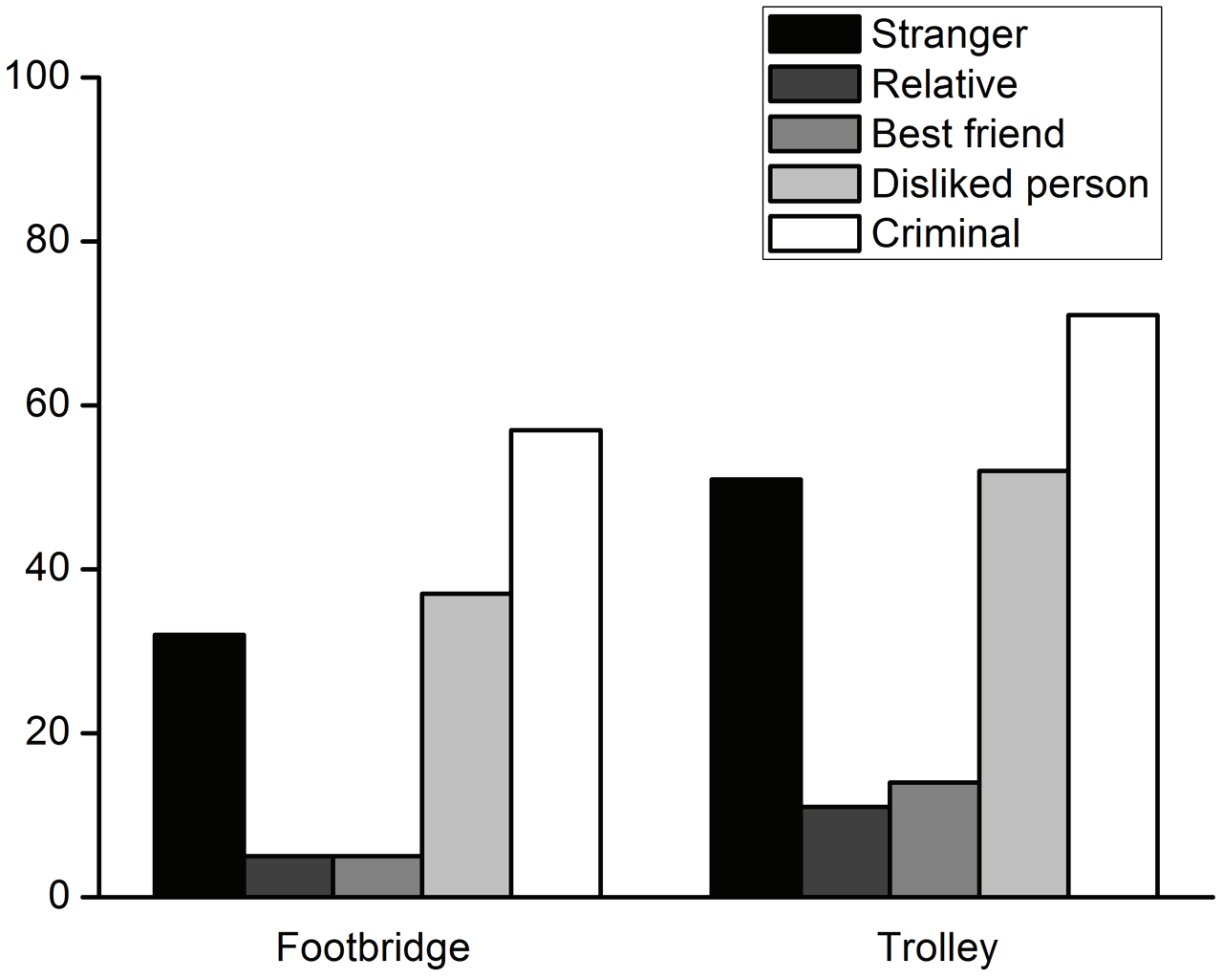
**Percentages of participants who decided to harm a lone potential victim to save five strangers from being hurt in Experiment 1**.

All of the participants answered the control questions correctly. The percentages of participants who decided to harm a specific person to save five strangers are shown in **Figure 1**. Binomial tests indicated that in the footbridge dilemma, when the lone potential victim was a stranger, a relative, a best friend, or a person whom the participant disliked, the percentage of the participants who decided to intervene was significantly lower than the chance level, *p* = 0.006, *p* < 0.001, *p* < 0.001, *p* = 0.046, respectively. There was no significant difference between the chance level and the percentage of participants who decided to harm a criminal, *p* = 0.321. In the trolley dilemma, binomial tests showed that the percentage of participants deciding to harm a relative or a best friend was also significantly lower than the chance level, *p*s < 0.001. The percentage of participants who decided to harm a stranger or person they disliked was comparable to the chance level, *p* = 1.000, *p* = 0.084, respectively. In addition, a large proportion of participants chose to sacrifice a criminal to save other people, *p* = 0.001.

Participants’ moral judgments involving each lone potential victim were compared using McNemar tests. The results of the footbridge dilemmas are shown in **Table 2**. Generally, of the participants who did not provide similar responses to each two-victim comparison, significantly more participants chose to harm one victim but not the other except for the relative vs. best friend and disliked person vs. stranger comparisons. In the trolley dilemma, similar results were obtained (**Table 3**).

**Table 2:** Comparisons of participants’ responses to different lone potential victim in the footbridge dilemma in Experiment 1.

	Percentages of participants who did and did not provide similar responses to each comparison	Of the participants who did not provide similar responses, number of participants choosing to harm the former and the latter	McNemar test for participants who did not provide similar responses
Relative vs. Stranger	72% vs. 28%	0 vs. 18	*p* < 0.001
Relative vs. Best friend	100% vs. 0%	0 vs. 0	*p* = 1.000
Relative vs. Disliked person	68% vs. 32%	0 vs. 21	*p* < 0.001
Relative vs. Criminal	48% vs. 52%	0 vs. 34	*p* < 0.001
Best friend vs. Stranger	72% vs. 28%	0 vs. 18	*p* < 0.001
Best friend vs. Disliked person	68% vs. 32%	0 vs. 21	*p* < 0.001
Best friend vs. Criminal	48% vs. 52%	0 vs. 34	*p* < 0.001
Disliked person vs. Stranger	86% vs. 14%	6 vs. 3	*p* = 0.508
Disliked person vs. Criminal	77% vs. 23%	1 vs. 14	*p* = 0.001
Criminal vs. Stranger	72% vs. 28%	17 vs. 1	*p* < 0.001

**Table 3:** Comparisons of participants’ responses to different lone potential victim in the trolley dilemma in Experiment 1.

	Percentages of participants who did and did not provide similar responses to each comparison	Of the participants who did not provide similar responses, number of participants choosing to harm the former and the latter	McNemar test for participants who did not provide similar responses
Relative vs. Stranger	57% vs. 43%	1 vs. 27	*p* < 0.001
Relative vs. Best friend	97% vs. 3%	0 vs. 2	*p* = 0.500
Relative vs. Disliked person	58% vs. 42%	0 vs. 27	*p* < 0.001
Relative vs. Criminal	40% vs. 60%	0 vs. 39	*p* < 0.001
Best friend vs. Stranger	60% vs. 40%	1 vs. 25	*p* < 0.001
Best friend vs. Disliked person	62% vs. 38%	0 vs. 25	*p* < 0.001
Best friend vs. Criminal	43% vs. 57%	0 vs. 37	*p* < 0.001
Disliked person vs. Stranger	86% vs. 14%	5 vs. 4	*p* = 1.000
Disliked person vs. Criminal	82% vs. 18%	0 vs. 12	*p* < 0.001
Criminal vs. Stranger	80% vs. 20%	13 vs. 0	*p* < 0.001

Experiment 1 indicated that adults made different moral judgments for individuals with whom they had different relationships. When the lone potential victims with whom they had a positive relationship were involved, participants preferred less utilitarian moral judgments. This result is consistent with previous studies (Bleske-Rechek et al., 2010; Tassy et al., 2013). Confronted with the lone potential victims with whom participants had negative relationships, participants showed a utilitarian tendency in their moral judgments. This finding indicates that apparent unfairness exists in adults’ relationship-based moral judgments. However, several issues remain to be clarified. First, it is not clear whether adults’ moral judgments involving specific individuals correspond to their relationships with the individuals. Second, it is also unknown whether their moral judgments involving specific individuals approximate their familiarity with the individuals or their affective relatedness to the individuals. In addition, the moral judgment pattern found in Experiment 1 must be confirmed with classic moral dilemmas in a large sample.

## Experiment 2

Experiment 2 explored whether there was latent fairness in adults’ relationship-based moral judgments. The extent to which adults were willing to protect a lone potential victim was compared to the extent to which they were familiar with and affectively related to the victim. A large sample of participants was tested with classic moral dilemmas.

### Participants

Three hundred six college students participated in the experiment: 133 males and 173 females. Their ages ranged from 18.05 to 24.28 years (*M* = 19.67, *SD* = 0.95). In a within-subject design, each participant responded to five test questions in a footbridge and a trolley dilemma. The stranger-conditioned questions were asked first because participants’ answers to these questions served as their baseline performance. The other questions and the two dilemmas were presented to the participants in a counterbalanced order (Latin square). Moreover, participants were asked to rate the extent to which they were familiar with and liked a specific potential victim. The experiment was approved by the Research Ethics Board of Department of Psychology of Capital Normal University. Informed written consent was obtained from all of the participants.

### Materials and Procedure

#### Moral Judgments

The moral dilemmas reported by Hauser et al. (2007) were used to assess participants’ moral judgments. A minor revision that was made in the present experiment was that participants served as the intervention actors in the dilemmas. For example, in the original footbridge dilemma, the intervention actor was a protagonist Frank. The intervention actors in the present experiment were the participants. Therefore, “Frank” was replaced by “you” in the moral dilemma below. Anonymous questionnaires were presented to participants. After reading each dilemma, participants were asked to indicate whether they should harm a specific individual to save five strangers first and then to rate their choices on a rating scale from 1 (very much should not do it), 2 (moderately should not do it), 3 (slightly should not do it), 4 (slightly should do it), 5 (moderately should do it) to 6 (very much should do it).

The footbridge dilemma (Hauser et al., 2007, p. 18)

You are on a footbridge over the train tracks. You know trains and can see that the one approaching the bridge is out of control. On the track under the bridge there are five people; the banks are so steep that they will not be able to get off the track in time. You know that the only way to stop an out-of-control train is to drop a very heavy weight into its path. But the only available, sufficiently heavy weight is a large man wearing a backpack, also watching the train from the footbridge. You can shove the man with the backpack onto the track in the path of the train, killing him; or you can refrain from doing this, letting the five die.

Test question 1: What should you do? Shove the man or not shove the man? Please choose from 1 (very much should not do it) to 6 (very much should do it). Test questions 2–5 were the same as test question 1 except that the lone potential victim was a relative, a best friend, a person the participant disliked and a criminal, respectively. For example, test question 2 was “If the man is your relative, shove the man or not shove the man? Please choose from 1 (very much should not do it) to 6 (very much should do it).” Therefore, in the footbridge dilemma, the lone potential victim was the one to shove.

The trolley dilemma (Hauser et al., 2007)

Minor revisions were made in the trolley dilemma so that the scenario was parallel to that in Experiment 1. In the trolley dilemma, participants could throw a switch to turn the train onto another track. However, the action would cause the death of a man standing on the other track. Participants were asked similar test questions. Therefore, in the trolley dilemma, the lone potential victim was the one who stood alone on the alternative track.

#### Rating of Relationships

Participants were asked to rate their familiarity with each specific potential victim on a rating scale from 1 (very unfamiliar with the person), 2 (moderately unfamiliar with the person), 3 (slightly unfamiliar with the person), 4 (slightly familiar with the person), 5(moderately familiar with the person) to 6 (very familiar with the person). Similarly, they were also required to rate their affective relatedness to the victim on a rating scale from 1 (very much dislike the person), 2 (moderately dislike the person), 3 (slightly dislike the person), 4 (slightly like the person), 5 (moderately like the person) to 6 (very much like the person). Anonymous questionnaires were used to measure participants’ relationships with the specific potential victims.

### Results and Discussion

#### Moral Judgments Involving Lone Potential Victims with Different Relationships with the Participants

**FIGURE 2:**
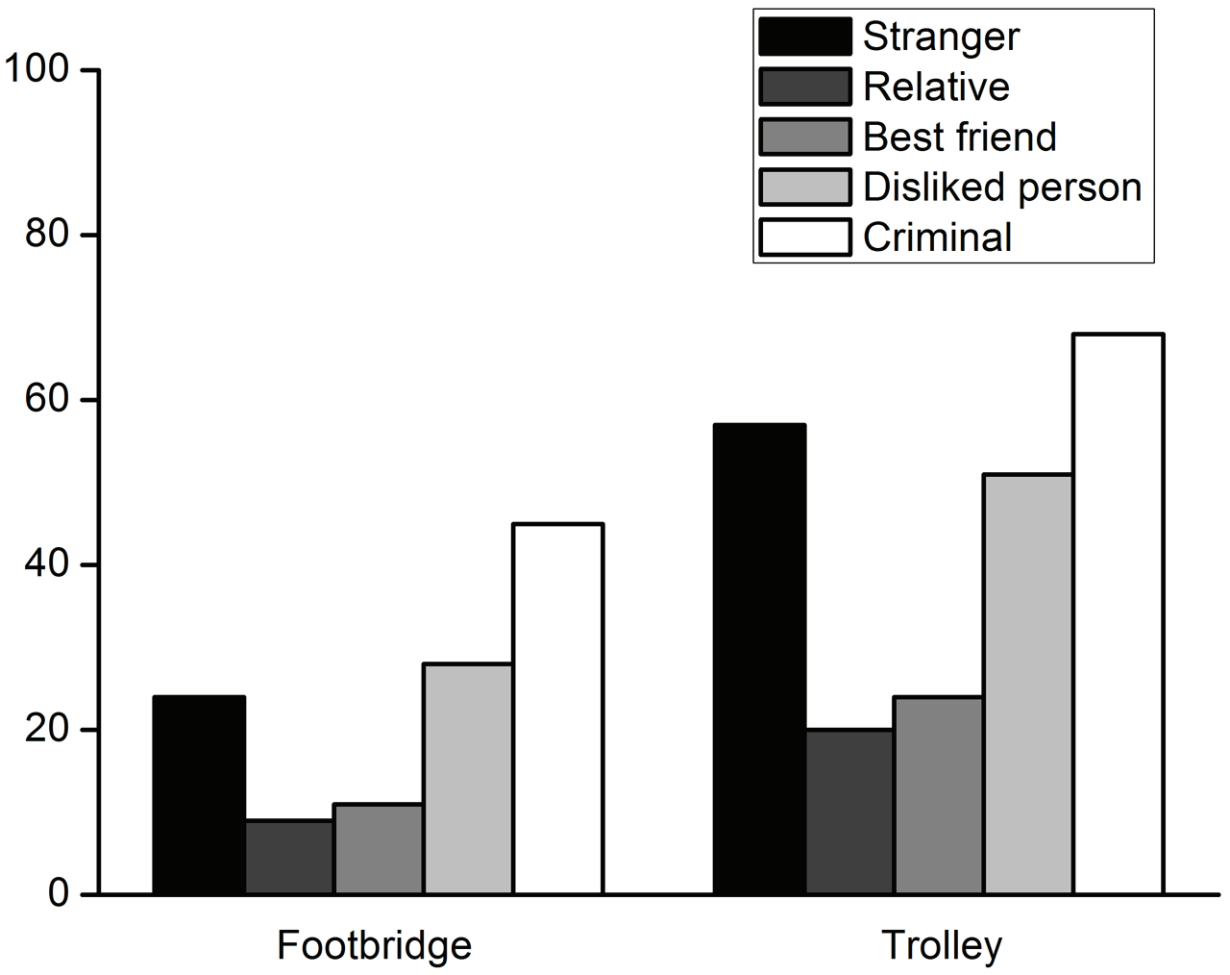
**Percentages of participants who decided to harm a lone potential victim to save five strangers from death in Experiment 2**.

The percentages of participant who decided to harm a specific person are shown in **Figure 2**. The percentages were compared to the chance level using binomial tests. In the footbridge dilemma, when the lone potential victim was a relative, a best friend, a disliked person, or a stranger, the percentage of participants deciding to harm the person was significantly lower than the chance level, *p*s < 0.001. There were no significant differences between the percentage of participants who were willing to harm a criminal and the chance level, *p* = 0.076. In the trolley dilemma, the percentage of participants deciding to harm a relative or best friend was also significantly lower than the chance level, *p*s < 0.001. The percentage of participants choosing to harm a disliked person was similar to the chance level, *p* = 0.864. The majority of participants were willing to sacrifice a criminal, *p* < 0.001. Therefore, the results were similar to those of Experiment 1 except that the percentage of participants who decided to harm a stranger was significantly above the chance level in the trolley dilemma*, p* = 0.019.

Participants’ moral judgments involving each specific potential victim were compared using McNemar tests. The results of the footbridge and trolley dilemmas are shown in **Tables 4** and **5**. The results resembled those of Experiment 1. However, some differences were found. In the footbridge dilemma, of the participants who made different decisions in the relative vs. best friend comparison, more participants were willing to sacrifice the best friend. Of the participants who made different decisions when a person they disliked and a stranger was involved, more participants chose to harm the person they disliked. In the trolley dilemma, more participants were willing to sacrifice a best friend compared to a relative, and a stranger compared to a disliked person.

**Table 4:** Comparisons of participants’ responses to different lone potential victim in the footbridge dilemma in Experiment 2.

	Percentages of participants who did and did not provide similar responses to each comparison	Of the participants who did not provide similar responses, number of participants choosing to harm the former and the latter	McNemar test for participants who did not provide similar responses
Relative vs. Stranger	84% vs. 16%	2 vs. 46	*p* < 0.001
Relative vs. Best friend	98% vs. 2%	0 vs. 6	*p* = 0.031
Relative vs. Disliked person	80% vs. 20%	2 vs. 60	*p* < 0.001
Relative vs. Criminal	64% vs. 36%	1 vs. 110	*p* < 0.001
Best friend vs. Stranger	84% vs. 16%	5 vs. 43	*p* < 0.001
Best friend vs. Disliked person	80% vs. 20%	4 vs. 56	*p* < 0.001
Best friend vs. Criminal	64% vs. 36%	3 vs. 106	*p* < 0.001
Disliked person vs. Stranger	88% vs. 12%	25 vs. 11	*p* = 0.030
Disliked person vs. Criminal	82% vs. 18%	2 vs. 53	*p* < 0.001
Criminal vs. Stranger	77% vs. 23%	67 vs. 2	*p* < 0.001

**Table 5:** Comparisons of participants’ responses to different lone potential victim in the trolley dilemma in Experiment 2.

	Percentages of participants who did and did not provide similar responses to each comparison	Of the participants who did not provide similar responses, number of participants choosing to harm the former and the latter	McNemar test for participants who did not provide similar responses
Relative vs. Stranger	58% vs. 42%	7 vs. 120	*p* < 0.001
Relative vs. Best friend	93% vs. 7%	4 vs. 17	*p* = 0.007
Relative vs. Disliked person	66% vs. 34%	5 vs. 99	*p* < 0.001
Relative vs. Criminal	48% vs. 52%	7 vs. 153	*p* < 0.001
Best friend vs. Stranger	63% vs. 37%	6 vs. 106	*p* < 0.001
Best friend vs. Disliked person	71% vs. 29%	4 vs. 85	*p* < 0.001
Best friend vs. Criminal	54% vs. 46%	4 vs. 137	*p* < 0.001
Disliked person vs. Stranger	83% vs. 17%	17 vs. 36	*p* = 0.013
Disliked person vs. Criminal	76% vs. 24 %	10 vs. 62	*p* < 0.001
Criminal vs. Stranger	80% vs. 20%	47 vs. 14	*p* < 0.001

**FIGURE 3:**
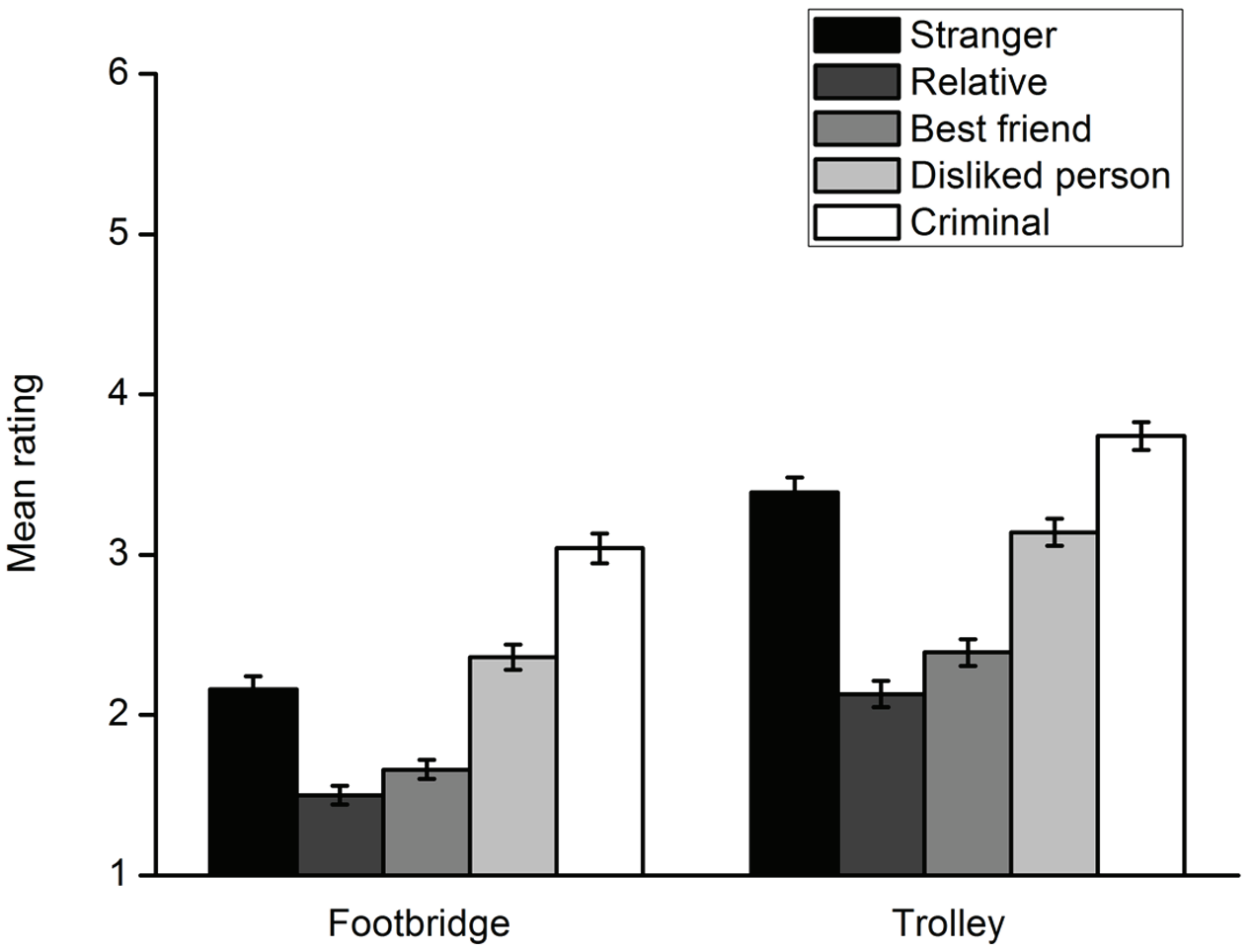
**Participants’ rating of their willingness to harm a lone potential victim to save five strangers from death in Experiment 2**. Error bars represent standard error.

Participants’ ratings of their willingness to harm a specific potential victim are shown in **Figure 3**. A 5 (victim type) × 2 (dilemma type) repeated measures ANOVA was conducted. A significant interaction effect between the two factors was found, *F*(4,302) = 11.44, *p* < 0.001, η^2^ = 0.132. First, a repeated measures ANOVA with the type of victim as the within-subject factor was carried out for each moral dilemma. In the footbridge dilemma, a main effect of the victim type was observed, *F*(4,302) = 67.98, *p* < 0.001, η^2^ = 0.474. Multiple comparisons with Bonferroni adjustment showed that there were significant differences between rating scores for any two potential victims, *p*s < 0.05. Participants were least willing to harm a relative and most willing to harm a criminal. In the trolley dilemma, there was also a main effect of the victim type, *F*(4,302) = 65.71, *p* < 0.001, η^2^ = 0.465. Multiple comparisons with Bonferroni adjustment indicated significant differences between rating scores for any two potential victims, *p*s < 0.05. The results were similar to those of the footbridge dilemma except that participants were more willing to sacrifice a stranger than a disliked person. Second, a *t* test was conducted for each lone potential victim to compare participants’ moral judgments in the footbridge and trolley dilemmas. The results indicated that participants decided to sacrifice a lone potential victim more unwillingly in the footbridge dilemma than they did in the trolley dilemma, regardless of the type of the victim. For the relative, *t*(305) = -8.13, *p* < 0.001, *d* = 0.480. For the best friend, *t*(305) = -8.70, *p* < 0.001, *d* = 0.511. For disliked person, *t*(305) = -9.16, *p* < 0.001, *d* = 0.521. For the criminal, *t*(305) = -7.40, *p* < 0.001, *d* = 0.425. For the stranger, *t*(305) = -12.28, *p* < 0.001, *d* = 0.706.

#### The Consistency between Moral Judgments Involving a Lone Potential Victim and Relationships with the Victim

**FIGURE 4:**
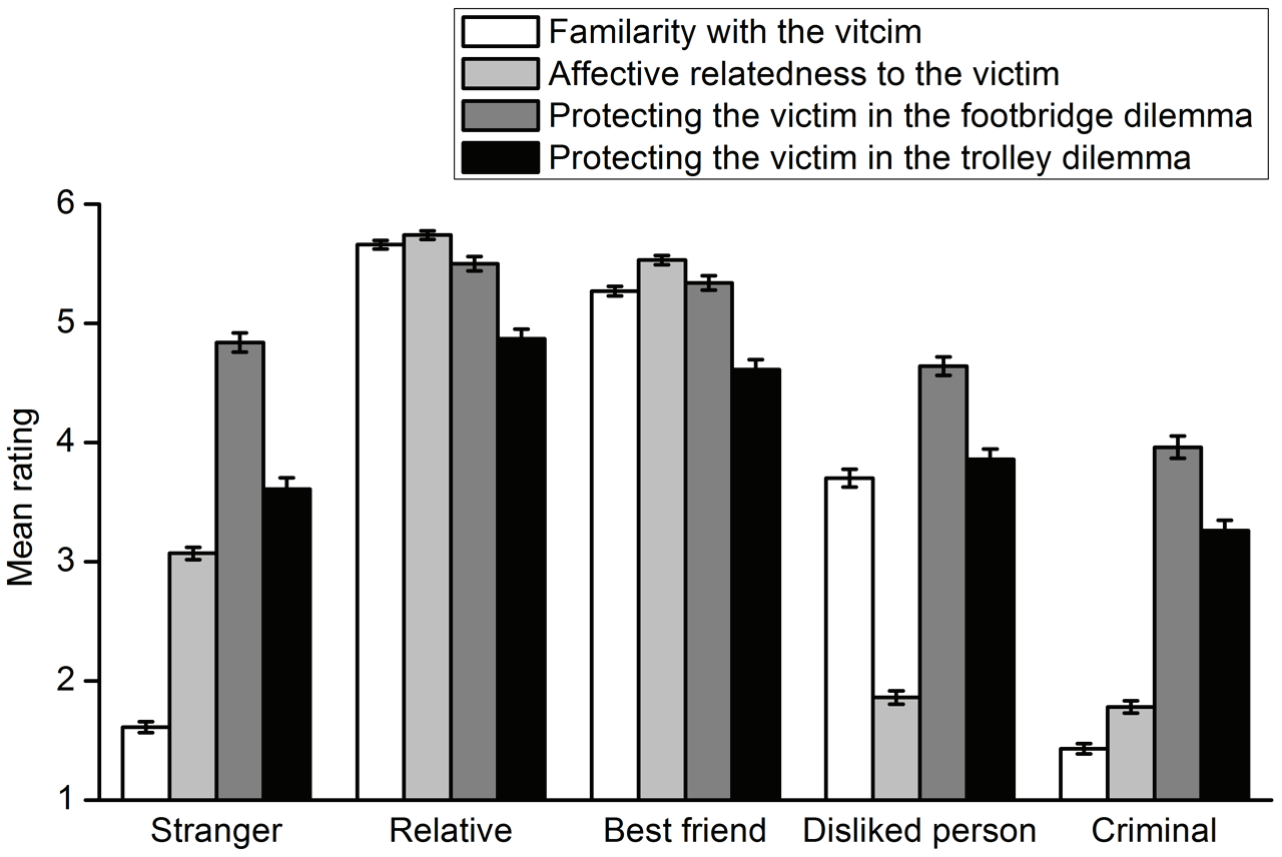
**Extent to which participants are familiar with, like and protect a lone potential victim in the footbridge and trolley dilemmas in Experiment 2**. Error bars represent standard error.

To investigate whether adults’ moral judgments involving a specific potential victim matched their relationships with the victim, their rating of willingness to harm a specific potential victim was reverse-scored. Thus, the reverse rating scores represented the extent to which adults were willing to protect the potential victim, i.e., deontological moral judgments. The extent to which participants are familiar with, like and protect a lone potential victim in the footbridge and trolley dilemmas are shown in **Figure 4**. A 5 (victim type) × 3 (rating type: familiarity, affective relatedness, willingness to protect a lone potential victim in the footbridge dilemma) repeated measures ANOVA indicated a significant interaction effect of the two factors, *F*(8,297) = 255.16, *p* < 0.001, η^2^ = 0.873. Thus, a repeated measures ANOVA with the rating type as the within-subject factor was carried out for each lone potential victim. For the relative, the main effect of rating type was significant, *F*(2,304) = 8.29, *p* < 0.001, η^2^ = 0.052. Multiple comparisons with Bonferroni adjustment showed that participants’ rating scores of their willingness to protect the relative were significantly lower than those of familiarity with and affective relatedness to their relative, *p* = 0.048, *p* = 0.001, respectively. For the best friend, a significant main effect was also found, *F*(2,304) = 24.09, *p* < 0.001, η^2^ = 0.137. Multiple comparisons indicated that participants’ rating scores of their willingness to protect the best friend were significantly lower than those of affective proximity to the best friend (*p* = 0.016) but not those of familiarity with the person (*p* = 0.868). When the lone potential victim was a person whom the participant disliked, the main effect of rating type was significant, *F*(2,303) = 510.83, *p* < 0.001, η^2^ = 0.771. However, multiple comparisons demonstrated that participants’ ratings of their willingness to protect the person were significantly higher than their ratings of familiarity with and affective relatedness to the person, *p*s < 0.001. Similar main effects were observed for both the criminal [*F*(2,304) = 338.37, *p* < 0.001, η^2^ = 0.690] and the stranger [*F*(2,304) = 674.43, *p* < 0.001, η^2^ = 0.816]. Participants’ ratings of their willingness to protect the criminal were significantly higher than those of their familiarity with and affective relatedness to the criminal (*p*s < 0.001). The results were the same for the stranger (*p*s < 0.001). A 5 (victim type) × 3 (rating type: familiarity, affective relatedness, willingness to protect a lone potential victim in the trolley dilemma) repeated measures ANOVA was then conducted. The results were generally the same as those discussed above. However, participants’ ratings of their willingness to protect the best friend were significantly lower than those of familiarity with and affective proximity to the person, *p*s < 0.001. In addition, their deontological moral judgment ratings concerning the person they disliked were significantly higher than their affective relatedness to the person (*p* < 0.001) but not their familiarity with the person (*p* = 0.407).

**FIGURE 5:**
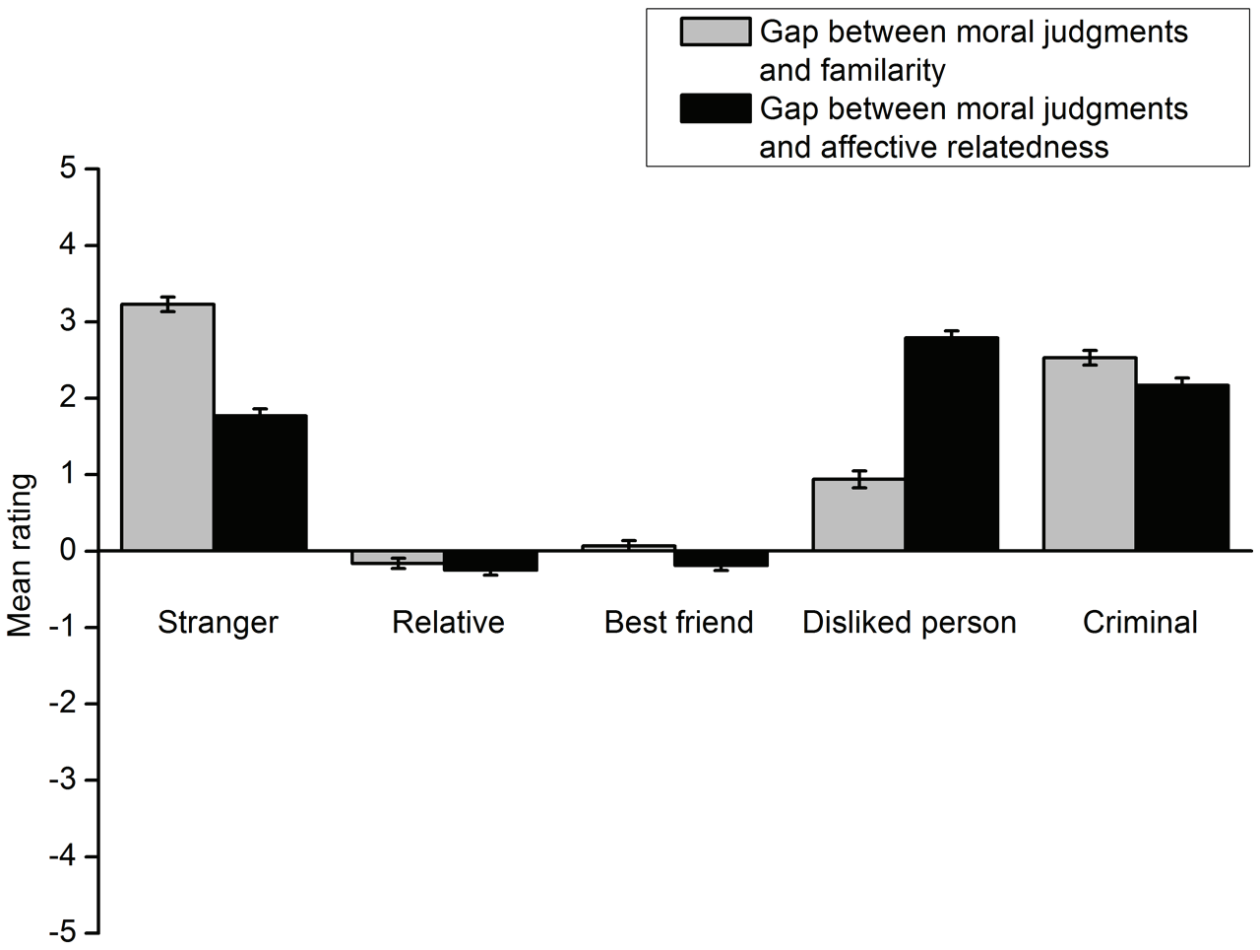
**Gaps between the extent to which participants are willing to protect a lone potential victim in the footbridge dilemma and the extent to which they are familiar with and like the victim in Experiment 2**. Error bars represent standard error.

**FIGURE 6:**
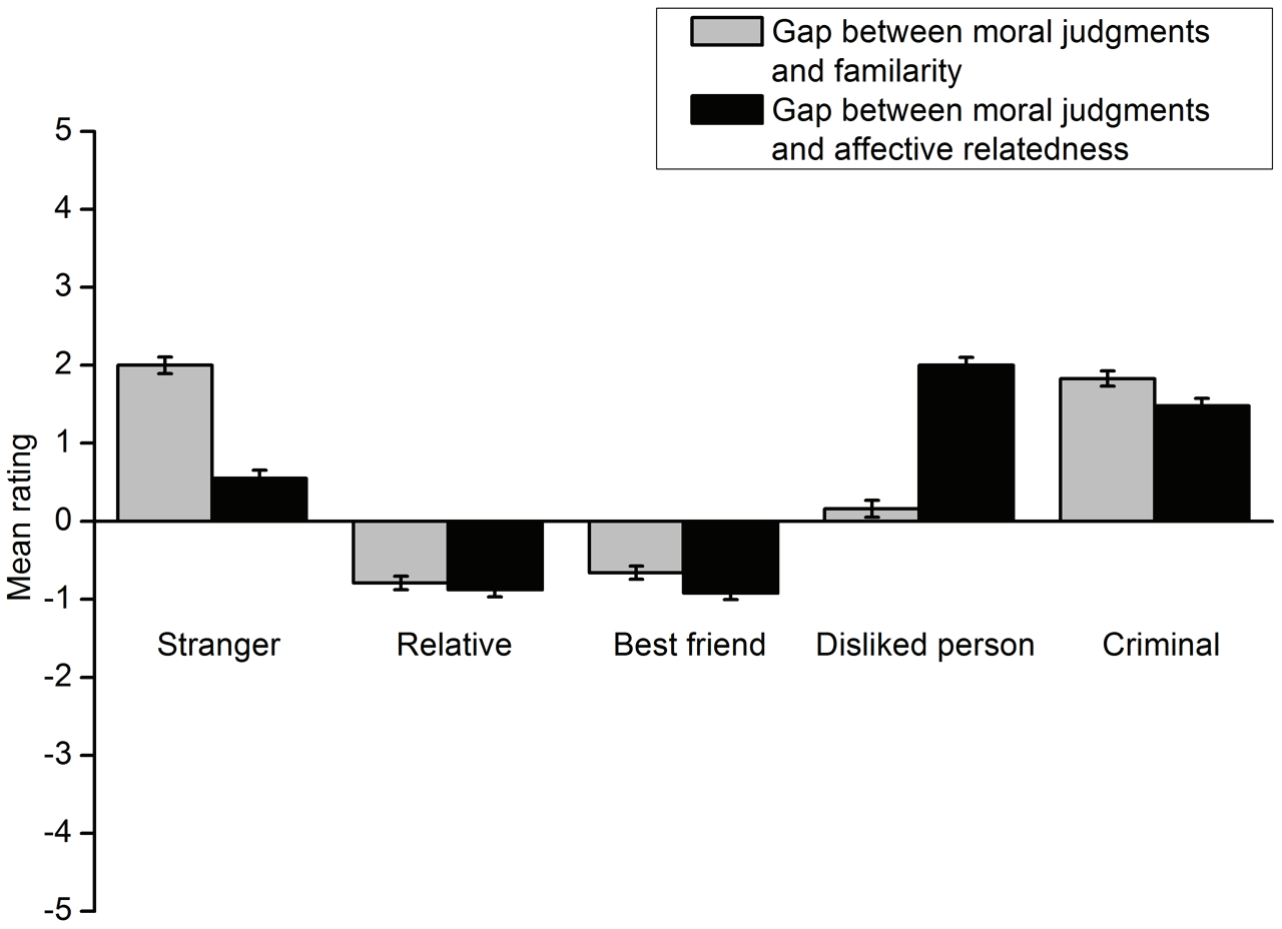
**Gaps between the extent to which participants are willing to protect a lone potential victim in the trolley dilemma and the extent to which they are familiar with and like the victim in Experiment 2**. Error bars represent standard error.

The results showed that participants’ deontological moral judgments involving a specific individual did not match their familiarity with or affective relatedness to the individual. The gap between participants’ willingness to protect a specific individual and familiarity with the individual was calculated by subtracting the rating score for the latter from that for the former. The gap between their willingness to protect a specific individual and affective relatedness to the individual was computed in the same manner. The results of the footbridge dilemma are shown in **Figure 5**. A 5 (victim type) × 2 (gap type) repeated measures ANOVA indicated a significant interaction effect of the two factors, *F*(4,301) = 204.72, *p* < 0.001, η^2^ = 0.731. A *t* test was conducted for each type of lone potential victim to compare the two gaps. Participants’ ratings of their willingness to protect the relative more closely approached the ratings of their familiarity with the person, *t*(305) = 2.56, *p* = 0.011, *d* = 0.162. Similar results were obtained for the best friend [*t*(305) = 6.85, *p* < 0.001, *d* = 0.395] and the person they disliked [*t*(304) = -19.87, *p* < 0.001, *d* = 1.155]. However, for the criminal and the stranger, participants’ deontological moral judgment ratings more closely approached their ratings of affective relatedness to these individuals [criminal: *t*(305) = 6.27, *p* < 0.001, *d* = 0.365; stranger: *t*(305) = 24.37, *p* < 0.001, *d* = 1.397].The results for the trolley dilemma are shown in **Figure 6**. All analyses and statistical results were identical to those of the footbridge dilemma.

In addition, the gaps between moral judgments and familiarity or affective relatedness in the footbridge dilemma were compared to those in the trolley dilemma. When the lone potential victim was a relative, the gap between the extent to which participants protected the person and the extent to which they were familiar with the person in the footbridge dilemma was significantly smaller than that in the trolley dilemma, *t*(305) = 8.13, *p* < 0.001, *d* = 0.475. The results were the same for the gap between the extent to which participants protected the relative and the extent to which they liked the person, *t*(305) = 8.13, *p* < 0.001, *d* = 0.476. When a best friend was involved, the gap between participants’ willingness to protect the person and their familiarity with the person in the footbridge dilemma was also significantly smaller than that in the trolley dilemma, *t*(305) = 8.70, *p* < 0.001, *d* = 0.506. The same results were obtained for the gap between deontological moral judgments involving the best friend and affective relatedness to the person, *t*(305) = 8.70, *p* < 0.001, *d* = 0.507. When the lone potential victim was a stranger, the gap between participants’ willingness to protect the person and their familiarity with the person in the footbridge dilemma was significantly larger than that in the trolley dilemma, *t*(305) = 12.83, *p* < 0.001, *d* = 0.706. The results were the same for the gap between deontological moral judgments involving the stranger and their affective relatedness to the person, *t*(305) = 12.83, *p* < 0.001, *d* = 0.701. Similarly, the gap between participants’ willingness to protect a lone potential victim and their familiarity with the person in the footbridge dilemma was also significantly larger than that in the trolley dilemma when the person was a disliked person [*t*(305) = 9.16, *p* < 0.001, *d* = 0.521] or a criminal [*t*(305) = 7.40, *p* < 0.001, *d* = 0.425]. The same results were obtained for the gap between participants’ deontological moral judgments involving a lone potential victim and their affective relatedness to the person when the person was a disliked person [*t*(304) = 9.17, *p* < 0.001, *d* = 0.528] or a criminal [*t*(305) = 7.40, *p* < 0.001, *d* = 0.419]. All the differences in the gaps between the footbridge and the trolley dilemma were due to participants’ greater willingness to protect the lone potential victim in the footbridge dilemma. As previous studies indicate, the finding reflects the contact principle.

Experiment 2 replicated the result that adults made different moral judgments for individuals with whom they had different relationships. More importantly, Experiment 2 illustrated that adults’ moral judgments involving specific individuals did not match their relationships with the individuals. How much they consider the good of the lone potential victim does not depend on how familiar they are with or how affectively they are related to the person. For relatives and best friends, adults’ moral judgments were less deontological relative to their relationships with these individuals. However, their deontological moral judgments involving people they disliked, criminals and strangers were far beyond their relationships with these individuals. These findings suggest that there may be some latent fairness in adults’ relationship-based moral judgments. Furthermore, the extent to which adults made deontological moral judgments involving relatives, best friends and people they disliked more closely approximated their familiarity with these people, whereas their deontological moral judgments involving criminals and strangers more closely approximated their affective relatedness to these people. Therefore, cognitive and affective dimensions of relationships may play different roles in adults’ moral judgments involving different individuals. These results were obtained when the participants made moral judgments toward a variety of lone potential victims. It is not clear whether the results are similar when the participants only respond to one type of victim. Thus, Experiment 3 was carried out with the type of lone potential victims as the between-subject variable.

## Experiment 3

Experiment 3 aimed to further test the results of Experiments 1 and 2 with the type of lone potential victim as the between-subject variable. Different groups of participants were required to make moral judgments involving different potential victims in footbridge and trolley dilemmas.

### Participants

A total of 628 college students took part in this experiment. The participants were divided into five groups. Each group was asked to decide whether to harm a specific potential victim to save five strangers in footbridge and trolley dilemmas. In each group, half of the participants first responded to the footbridge dilemma. One hundred twenty-five participants responded to the stranger dilemmas: 60 males and 65 females. Their ages ranged from 17.15 to 26.33 years (*M* = 19.76, *SD* = 1.72). One hundred twenty participants made moral judgments in the relative dilemmas: 59 males and 61 females. Their ages ranged from 17.35 to 24.22 years (*M* = 19.68, *SD* = 1.29). One hundred eighteen participants made decisions in the best friend dilemmas: 54 males and 64 females. Their ages ranged from 17.55 to 26.09 years (*M* = 19.72, *SD* = 1.40). In the disliked person dilemmas, there were 136 participants. Half of them were males. Their ages ranged from 17.26 to 26.02 years (*M* = 20.01, *SD* = 1.51). In the criminal dilemmas, there were 129 participants: 67 males and 62 were females. Their ages ranged from 17.07 to 24.77 years (*M* = 20.03, *SD* = 1.71). There were no significant differences in ages between the five groups, *F*(4,623) = 1.46, *p* = 0.231, η^2^ = 0.009. The experiment was approved by the Research Ethics Board of Department of Psychology of Capital Normal University. Informed written consent was obtained from all of the participants.

### Materials and Procedure

#### Moral Judgments

The materials and procedure were similar to those of Experiment 2. One difference was that each group of participants only responded to moral dilemmas involving a specific potential victim. Take the relative footbridge dilemma for example.

The footbridge dilemma (Hauser et al., 2007, p. 18)

You are on a footbridge over the train tracks. You know trains and can see that the one approaching the bridge is out of control. On the track under the bridge there are five people; the banks are so steep that they will not be able to get off the track in time. You know that the only way to stop an out-of-control train is to drop a very heavy weight into its path. But the only available, sufficiently heavy weight is a large man wearing a backpack, also watching the train from the footbridge. And this man is your relative. You can shove your relative with the backpack onto the track in the path of the train, killing him; or you can refrain from doing this, letting the five die.

Participants were then asked test questions “What should you do? Shove your relative or not shove him? Please choose from 1 (very much should not do it) to 6 (very much should do it).”

The trolley dilemma (Hauser et al., 2007)

In the trolley dilemma, participants could throw a switch to turn the train onto another track, but the action would cause the death of a relative. They were then asked similar test questions.

#### Rating of Relationships

Each group of participants was asked to rate their familiarity with and affective relatedness to the specific potential victim on the same six-point scales used in Experiment 2.

### Results and Discussion

#### Moral Judgments Involving Lone Potential Victims with Different Relationships with the Participants

**FIGURE 7:**
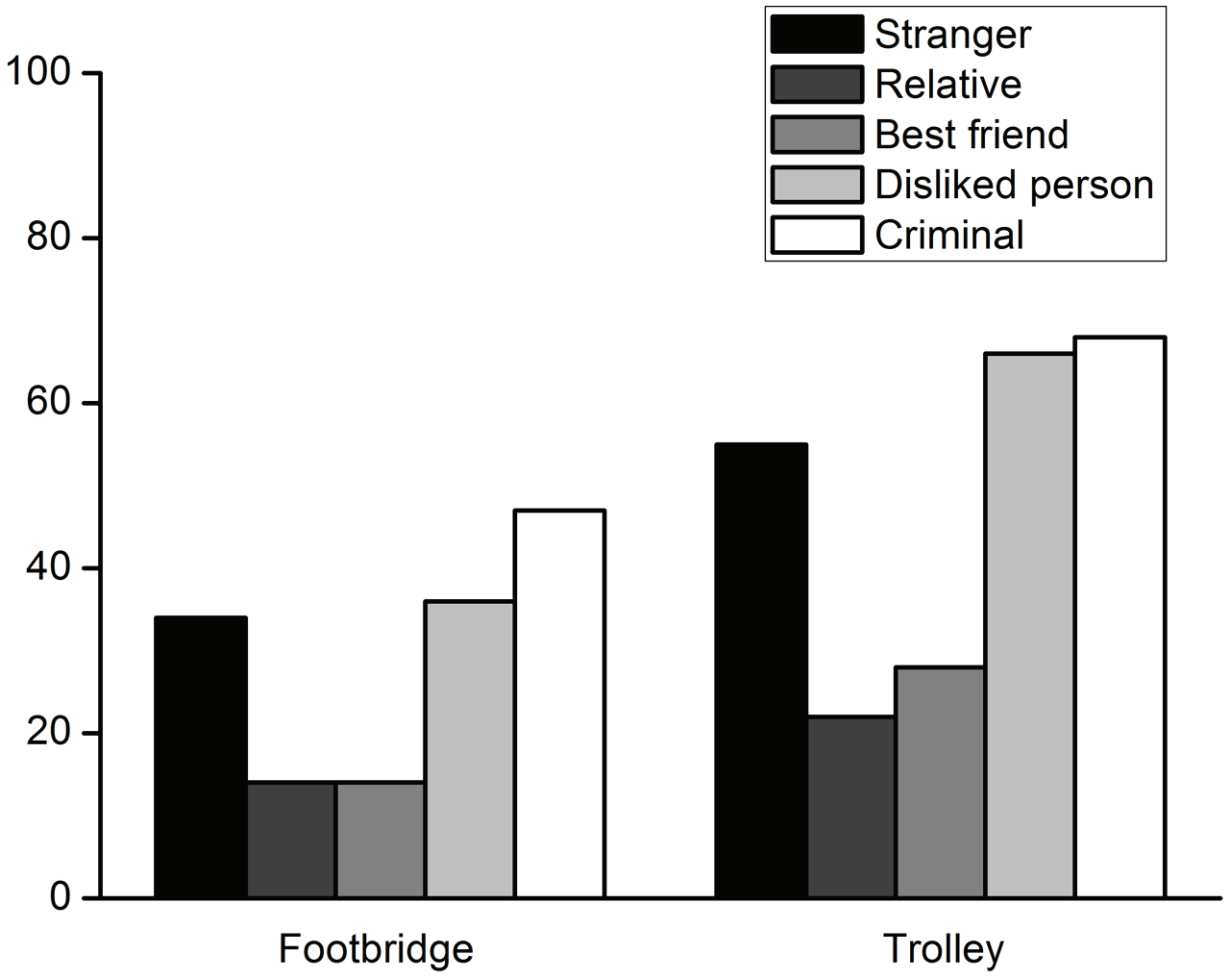
**Percentages of participants who decided to harm a lone potential victim to save five strangers from death in each group in Experiment 3**.

Each group’s percentage of participants choosing to harm the lone potential victim is shown in **Figure 7**. Each percentage was compared to the chance level with binomial tests. In the footbridge dilemma, the percentage of participants who decided to harm the lone potential victim was significantly lower than the chance level in the relative group (*p* < 0.001), best friend group (*p* < 0.001), disliked person group (*p* = 0.001) and stranger group (*p* < 0.001). For the criminal group, the percentage was not significantly different from the chance level, *p* = 0.597. In the trolley dilemma, the percentage of participants deciding to harm the lone potential victim was also significantly lower than the chance level in the relative group and best friend group, *p*s < 0.001. There were no significant differences between the percentage and the chance level in the stranger group, *p* = 0.283. The percentage of participants who were willing to sacrifice a disliked person or a criminal was significantly higher than chance level, *p*s < 0.001. The results were generally similar to those obtained in Experiments 1 and 2 except that more participants chose to sacrifice the disliked person in the trolley dilemma.

**FIGURE 8:**
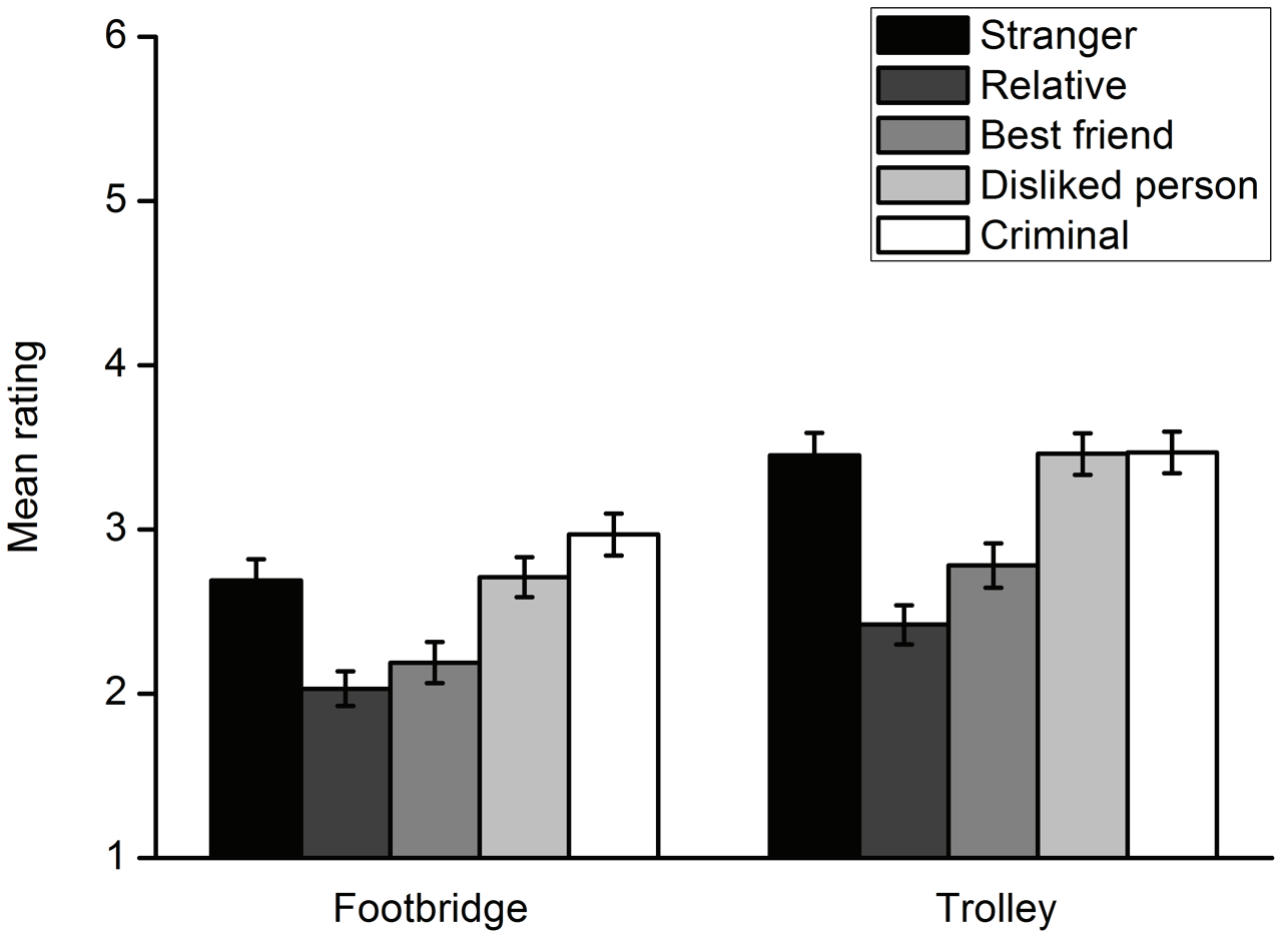
**Participants’ rating of their willingness to harm a lone potential victim to save five strangers from death in each group in Experiment 3**. Error bars represent standard error.

Each group’s rating of their willingness to harm a lone potential victim to save five strangers is shown in **Figure 8**. An ANOVA was conducted to explore the group differences in the footbridge dilemma. The effect of group was significant, *F*(4,623) = 10.13, *p* < 0.001, η^2^ = 0.061. *Post hoc* Bonferroni tests showed that the rating scores of the relative group were significantly lower than those of the disliked person (*p* = 0.001), criminal (*p* < 0.001) and stranger groups (*p* = 0.002) but not the best friend group (*p* = 1.000). The rating scores of the best friend group were also significantly lower than those of the disliked person (*p* = 0.028), criminal (*p* < 0.001) and stranger groups (*p* = 0.047). There were no significant differences in rating scores between the disliked, criminal and stranger group, *p*s = 1.000. The results were similar to those obtained for the trolley dilemma. Overall, adults made different moral judgments toward close individuals and those they were not close to.

#### The Consistency between Moral Judgments Involving a Lone Potential Victim and Relationships with the Victim

**FIGURE 9:**
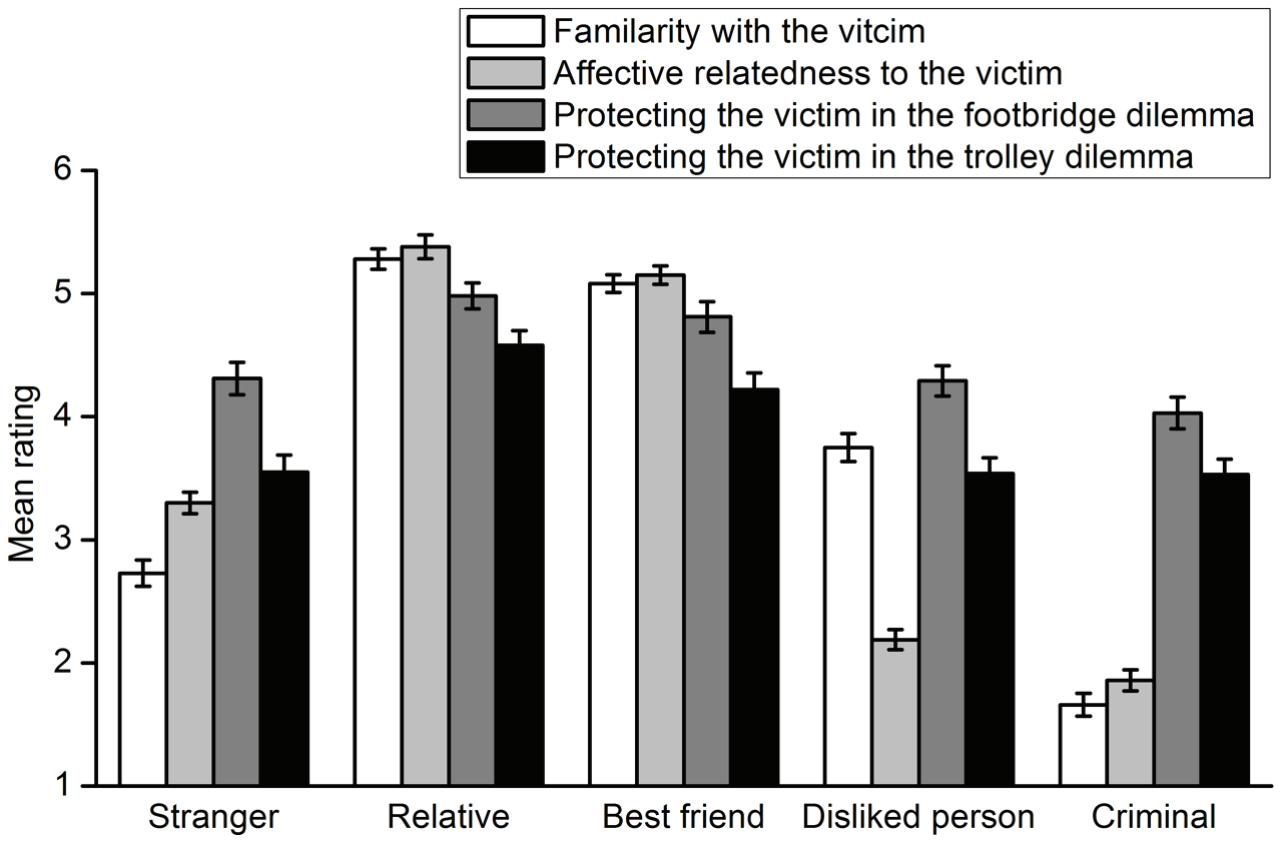
**Extent to which each group of participants are familiar with, like and protect a lone potential victim in the footbridge and trolley dilemmas in Experiment 3**. Error bars represent standard error.

Participants’ willingness to harm a lone potential victim was reverse-scored. The resultant scores represented the extent to which adults were willing to protect the lone potential victim, i.e., deontological moral judgments. The extent to which each group of participants is familiar with, likes and protects a lone potential victim in the footbridge and the trolley dilemma is shown in **Figure 9**. A 5 (group) × 3 (rating type: familiarity, affective relatedness, willingness to protect the lone potential victim in the footbridge dilemma) repeated measures ANOVA was performed. There was a significant interaction effect of the two factors, *F*(8,1246) = 73.40, *p* < 0.001, η^2^ = 0.320. Simple effect analysis was conducted with separate repeated measures ANOVA for each group. For the relative group, the effect of rating type was significant, *F*(2,118) = 4.38, *p* = 0.015, η^2^ = 0.069. Multiple comparisons with Bonferroni adjustment indicated that participants’ rating scores of their willingness to protect the relative were marginally significantly lower than those of their familiarity with the relative (*p* = 0.052) and significantly lower than those of their affective relatedness to the relative (*p* = 0.011). For the best friend group, the effect of rating type approached significance, *F*(2,116) = 3.05, *p* = 0.051, η^2^ = 0.050. Multiple comparisons found that participants’ rating scores of their willingness to protect the best friend were marginally significantly lower than those of their affective relatedness to the person (*p* = 0.057) but not those of their familiarity with the person (*p* = 0.181). There was a significant effect of rating type in the disliked person group, *F*(2,134) = 132.78, *p* < 0.001, η^2^ = 0.665. The rating scores of participants’ willingness to protect the disliked person were both significantly higher than those of their familiarity with (*p* = 0.004) and affective relatedness to the person (*p* < 0.001). The same effect was found for the criminal group, *F*(2,127) = 121.87, *p* < 0.001, η^2^ = 0.657. There were significant differences between the rating scores of their willingness to protect the criminal and those of their familiarity with the person (*p* < 0.001) or affective relatedness to the person (*p* < 0.001). For the stranger group, the effect of rating type was also significant, *F*(2,123) = 53.49, *p* < 0.001, η^2^ = 0.465. The extent to which participants were willing to protect a stranger was significantly higher than the extent to which they were familiar with (*p* < 0.001) and liked the stranger (*p* < 0.001). A 5 (group) × 3 (rating type: familiarity, affective relatedness, willingness to protect the lone potential victim in the trolley dilemma) repeated measures ANOVA was performed. The results were similar to those involving the footbridge dilemma. Generally, participants’ rating scores of their willingness to protect the lone potential victim was significantly different from those of their familiarity with and affective relatedness to the person in each group. However, participants’ rating scores of their willingness to protect the lone potential victim were comparable to those of their familiarity with the victim in the disliked person group, *p* = 0.719. The same result was obtained for the comparison of willingness to protect the lone potential victim and affective relatedness to the victim in the stranger group, *p* = 0.449. Overall, these results replicated those of Experiment 2.

**FIGURE 10:**
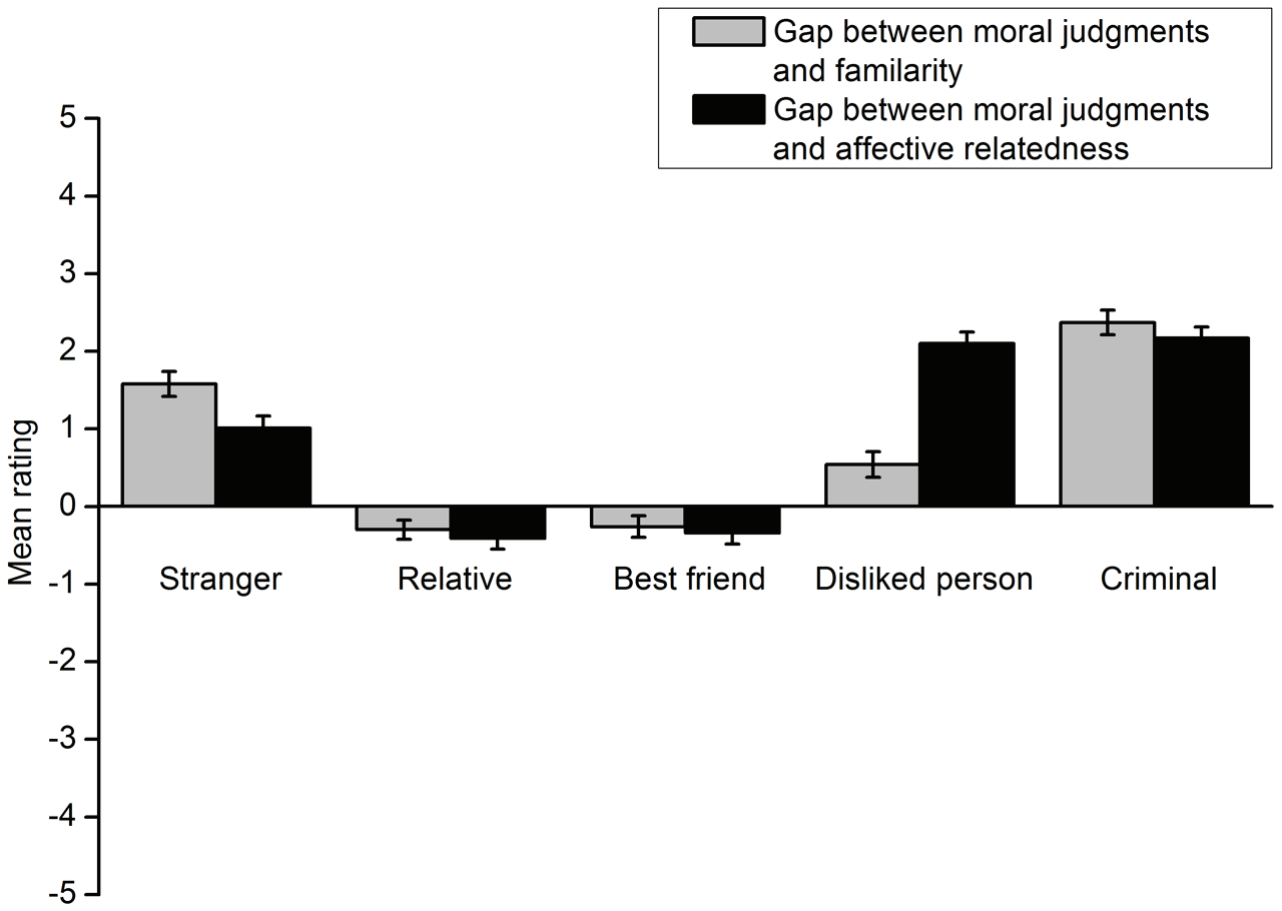
**Gaps between the extent to which each group of participants are willing to protect a lone potential victim in the footbridge dilemma and the extent to which they are familiar with and like the victim in Experiment 3**. Error bars represent standard error.

**FIGURE 11:**
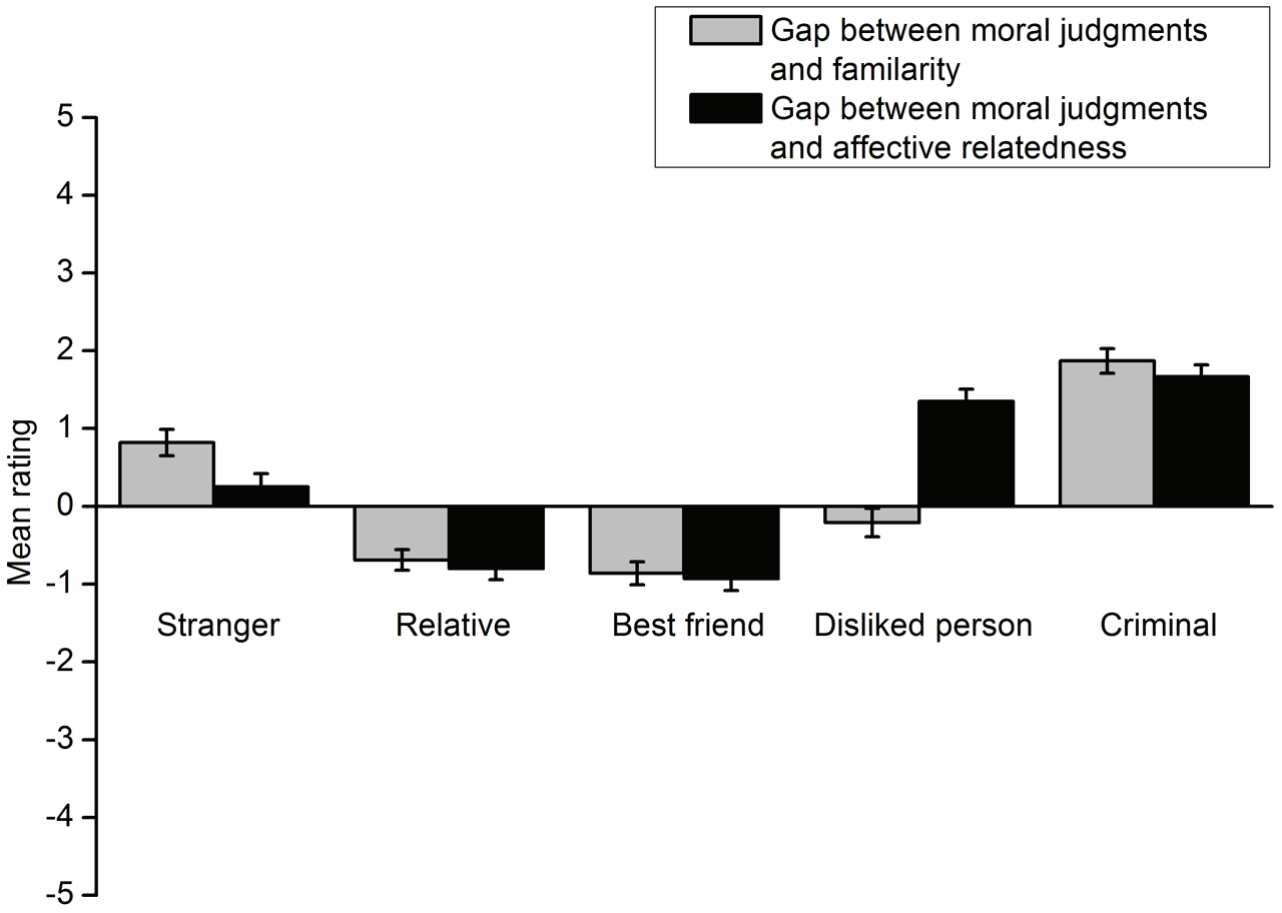
**Gaps between the extent to which each group of participants are willing to protect a lone potential victim in the trolley dilemma and the extent to which they are familiar with and like the victim in Experiment 3**. Error bars represent standard error.

The gaps between participants’ willingness to protect a lone potential victim and their relationships with the victim in each group were then analyzed. The results of the footbridge dilemma are shown in **Figure 10**. A 5 (group) × 2 (gap type) repeated measures ANOVA was carried out. The interaction effect was significant, *F*(4,623) = 83.21, *p* < 0.001, η^2^ = 0.348. A *t*-test was then conducted for each group. The gap between deontological moral judgments and familiarity with the lone potential victim was not significantly different from the gap between deontological moral judgments and affective relatedness to the victim in the relative group [*t*(119) = 1.51, *p* = 0.134, *d* = 0.142] and the best friend group [*t*(117) = 1.32, *p* = 0.190, *d* = 0.127]. For the disliked person group, participants’ willingness to protect the lone potential victim more closely approximated their familiarity with the victim, *t*(135) = -11.93, *p* < 0.001, *d* = 1.032. The opposite results were found for the criminal group [*t*(128) = 2.40, *p* = 0.018, *d* = 0.213] and the stranger group [*t*(124) = 6.18, *p* < 0.001, *d* = 0.547]. These two groups’ willingness to protect the lone potential victim more closely approximated their affective relatedness to the victim. The results of the trolley dilemma are shown in **Figure 11**. All the analyses and statistical results were similar to those obtained for the footbridge dilemma. Taken together, these results generally replicated those of Experiment 2 except that deontological moral judgments did not more closely approximate familiarity with the lone potential victim in extent rating for the relative and best friend groups.

Experiment 3 generally confirms the results of Experiments 1 and 2 with the victim type as the between-subject variable. First, the results indicate that adults’ relationship-based moral judgments are apparently unfair. Adults were more unwilling to harm a relative or best friend compared to a disliked person, a criminal or a stranger as indicated by the results of Experiments 1 and 2. The results may reflect adults’ different treatment to ingroup and outgroup members. The distinction between ingroup and outgroup is especially salient in collectivistic cultures (Triandis, 1993). Meanwhile, adults made similar choices toward the relative and the best friend, and they also treated the disliked person, the criminal and the stranger similarly. These results appear inconsistent with those of the previous experiments. Different situations induced by specific experimental designs may result in different responses. In the within-subject design, participants must make moral judgments toward different potential victims simultaneously. Participants are more likely to finely differentiate between one victim and another. In the between-subject design, participants only respond to a specific potential victim. Without comparison, their moral judgments involving a specific individual may be based on those involving a category of people. Therefore, individuals of the same category may be treated similarly. Second, the results indicate that latent fairness exists in adults’ relationship-based moral judgments. Adults’ moral judgments were less deontological relative to their close relationships with relatives and best friends, but more deontological relative to their distant relationships with disliked people, criminals and strangers. Finally, the results show that familiarity is more important in adults’ moral judgments involving familiar individuals, especial disliked people. Affective relatedness plays a more crucial role in their moral judgments involving unfamiliar individuals.

## General Discussion

Moral judgments are closely associated with moral behavior (Wu and Liu, 2014). However, moral judgments are not invariable. As one enters adulthood, one has contact with an increasing number of people, and one’s moral judgments are inevitably influenced by one’s relationships with others. Thus, understanding adults’ relationship-based moral judgments is theoretically important for explaining moral behavior in adulthood.

The present study demonstrated that adults made different moral judgments for individuals with whom they had different relationships. Specifically, when the lone potential victim was a close individual, including a relative and a best friend, adults opposed utilitarian moral judgments. Moreover, less utilitarian moral judgments were made for the relative than for the best friend in Experiments 1 and 2. The results are consistent with the findings of previous studies (Bleske-Rechek et al., 2010; Tassy et al., 2013). However, the present study also considered adults’ moral judgments involving individuals who were not close to them, i.e., people they disliked and criminals, and indicated utilitarian tendencies toward these individuals. Meanwhile, the results indicated that criminals were subjected to more utilitarian moral judgments than people adults disliked in Experiments 1 and 2. Therefore, adults display unfair treatment of people over the entire hierarchy of relationships. The apparent unfairness of adults’ moral judgments may be traced to early moral development. For example, children’s resource-allocation decisions vary according to the recipients (Moore, 2009). They treat their friends in the best manner and other people less well. The trajectories of adolescents’ prosocial behavior toward family members and friends also differ (Padilla-Walker et al., 2015). In addition, some studies show that the age of the lone potential victim influences adults’ moral judgments (Bleske-Rechek et al., 2010; Kawai et al., 2014). Adults are more willing to sacrifice an older person to save more people. It is suggested that adult’ moral judgments are unfair when confronted with people of different ages. Despite the unfairness, adults’ moral decisions were relatively conservative in the present study. Previous studies have shown that Chinese participants are less inclined to sacrifice one person to save more people than Western participants (Ahleniusa and Tannsjö, 2012; Gold et al., 2014).

A more prominent and new finding of the present study was that adults’ moral judgments involving a specific individual did not correspond to their relationship with the individual. For close individuals, adults’ moral judgments were less deontological relative to their familiarity with or positive affect toward these individuals. By contrast, for individuals they were not close to, adults made deontological choices to a larger extent relative to their unfamiliarity with or negative affect toward these individuals. The results thus provide evidence of latent fairness in adults’ relationship-based moral judgments. According to Kohlberg’s cognitive-development theory, there are six developmental stages of moral judgments (Kohlberg, 1963). One’s moral judgments develop from obedience to external power, e.g., from punishment and authority to internal ethical principles. Empirical studies also indicate that adults’ stage 5 and stage 6 scores are significantly higher than adolescents’ scores (Martin et al., 1977). Thus, adults have relatively mature moral judgments and are able to consider abstract principles such as fairness to some extent. Specifically, adults may have tried to prevent their moral judgments from being affected by their relationships with the lone potential victims in the present study, which resulted in the inconsistency between their moral judgments involving a specific potential victim and their relationships with the victim. Moreover, certain moral intuitions, including the foundation of fairness/reciprocity, are believed to have developed to guide moral judgments (Graham et al., 2009). The fairness intuition may motivate adults to adjust their moral judgments involving specific individuals. From the perspective of culture, unlike European American culture’s emphasis on individuality, Chinese culture underlines the harmony and interrelatedness of all individuals (Doan and Wang, 2010). The cultural characteristics may facilitate adults’ consideration of fairness in their moral decisions, especially when considering individuals with whom they have negative relationships. Consistent with this argument, both Experiments 2 and 3 demonstrated that the extent to which adults were willing to protect a disliked person or a criminal was substantially higher than the extent to which they were familiar with or liked the person.

Furthermore, the present study demonstrated that for familiar individuals, especially the disliked person, the extent to which adults made deontological moral judgments approximated the extent to which they were familiar with the individual. However, their deontological moral judgments involving unfamiliar individuals approximated their affective relatedness to the individuals. The results suggest that adults’ moral judgments involving familiar people depend more on their cognition about these people, whereas those involving unfamiliar people are more strongly influenced by their affect toward these people. Researchers also propose that certain moral judgments are guided by emotional processes, whereas others largely engage one’s cognitive processes (Greene et al., 2001, 2004). Based on Greene et al. (2001, 2004) perspectives, emotional responses are aroused at once in moral situations, but cognitive control may guide moral judgments when there is sufficient time or motivation (Conway and Gawronski, 2013). Cognitive and affective processes were elicited by specific situations in the studies by Greene et al. (2001, 2004) whereas they were driven by specific individuals in the present study. However, the results of the present study can also be explained by the perspectives discussed above. When facing moral dilemmas involving a specific individual, adults’ affect toward the individual may play an initial role in their moral judgments. However, with the motivation of latent fairness, adults may attempt to avoid being influenced by their affective relatedness to familiar individuals and, thus, rely more on their cognitive information when making moral judgments. Adults’ affective relatedness to unfamiliar individuals is the only available information for their moral judgments. Therefore, moral judgments involving unfamiliar individuals depend more on adults’ affective relatedness to these individuals. The varying importance of cognition and affect in moral judgments involving different individuals can be considered further evidence of latent fairness in adults’ moral judgments.

In sum, the present study reveals two sides of adults’ relationship-based moral judgments. Apparent unfairness exists in their relationship-based moral judgments. Nevertheless, when adults’ relationships with specific individuals are measured and taken into account, there is latent fairness in adults’ moral judgments. Moreover, moral judgments involving individuals with whom adults have different relationships have different cognitive and affective bases.

## Author Contributions

JH proposed the conception and designed the work; JH, YL, and JL performed the acquisition, analysis, and interpretation of data for the work; JH drafted the work; JH, YL, and JL revised the work for important intellectual content. All the authors finally approved the version to be published. All the authors agreed to be accountable for all aspects of the work in terms of ensuring that questions related to the accuracy or integrity of any part of the work are appropriately investigated and resolved.

## Conflict of Interest Statement

The authors declare that the research was conducted in the absence of any commercial or financial relationships that could be construed as a potential conflict of interest.
